# Massachusetts Dental Society

**Published:** 1889-07

**Authors:** 


### Reports of Society Meetings.

### MASSACHUSETTS DENTAL SOCIETY.

The semi-annual meeting of the Massachusetts Dental Society was held in Boston, June 5, 6, and 7, 1889.

*Wednesday, June 5.—Afternoon Session.*

### RESPONSES OF DELEGATES.

*Dr. George W. Lovejoy*, Montreal, Canada.—It affords me great pleasure to be with you at this meeting. I was at your meeting last year, and enjoyed myself very much indeed. The exhibit of dental appliances is a new feature, and is certainly of great benefit to all who attend.

*Dr. Magee*, St. Johns, New Brunswick.—I am a young member of the profession, and came here to learn. I am very glad to be with you, and thank Dr. Merriam and you all for the invitation to attend.

*Dr. B. H. Teague*, Aiken, South Carolina.—I am afraid Dr. Merriam has taken a liberty with me in asking me to make an address. I did not expect to be the speaker from South Carolina. The original speaker has either been engulfed in the floods or been kept at home on account of affliction; and yet South Carolina has never been called upon in the past but what she responded in some way or other,—right or wrong. It is with some feelings of intimidation that I am here among you. It was said down South that, "if such a little rebel as you get up there, some of those big Yankees, like Cooke, will overhaul you." I said I knew something about the Yankees; in fact, I had learned a great deal about them, for my father-in-law was a New-Hampshire-born man, and a practitioner of dentistry for many years. His name is familiar to a great many, and I must say that what success I have had in life has come from the teachings of that good old Yankee preceptor. Gentlemen, he was a shrewd old man. He did not exactly make me a student, but he claimed that I should stay with him three long years. I stayed the three years, and rather think the old gentleman got the best of me; but, by marrying his daughter, I got the best of him.

Now, sirs, my practice has been mostly among Northern people. I live at the little town called Aiken, visited by many of your unfortunate sick people. In the mouths of these people I have seen very little work that I would be ashamed of, and a great deal that has spurred on my ambition. I have not come here to show you anything great, but, as the gentleman said, to learn, and I hope that in the proceedings of your meeting I shall be filled with all I can digest.

*Dr. J. N. Crouse*, Chicago.—It affords me great pleasure to be in Boston again. It has been a number of years since I was so far East, as my recreation takes me westward. I am glad to see so many of my old friends again. A number I see around me looking as they did ten years ago, having grown a little gray, but just as smiling as ever, and they make you feel just as comfortable when they take your hand. I came a long way to this city, as one of the speakers said, to learn something. I get rusty at home, and then, when I get where I think I do not know as much as other practitioners, I strike out and come East, and pick up knowledge here and there, and get whatever I can. Even if it does not go to my head,

I can put it in my pocket and take it home with me. I tried to persuade some of my Western brothers to come with me, but could not prevail on them. I shall certainly carry back with me many new and profitable things to tell them when I go home. I brought with me a big satchel so that I could carry along anything that I got, and you need not be afraid of overloading me, for I could get a trunk, and I will be ready to receive anything that the New England and Massachusetts Dental Societies will be willing to give me.

*Dr. S. H. Guilford*, Philadelphia.—The city of Philadelphia (and, perhaps, I may say the State of Pennsylvania) sends greetings to the Massachusetts Dental Society in its present meeting. It also sends its well-wishes for its success. Personally, I regret very much indeed that there are so few representatives here from my own city. The meetings of your Society are well known all over the country. I know in Philadelphia it is a pleasure to read of your proceedings, published in our dental journals, and I know many more would be benefited if they had concluded to come to this meeting. Your Executive Committee have the happy faculty of bringing everything here, and doing good with it, so that when we come here we feel that we are not only to see you personally, but what you have been able to gather.

You have also added a new feature to your meetings, and that is, the idea of bringing the profession in contact with the manufacturer or first dealer. That, I think, has never been done before, and the credit surely belongs to this Society. It is an important matter, and brings many persons to your meeting. All interested in teaching young men in the colleges understand the importance of keeping abreast of the times, not only with regard to the theory and practice of dentistry, but also in the important matter of materials used in dentistry. The student must not only be taught to practise, but he needs to be put in direct communication with the manufacturer, and this is the first occasion where I have been able to gather such information. I wish you every success in the meeting.

*Dr. G. L. Chewning*, Fredericksburg, Virginia.—There are moments in life when the emotions of the heart cannot be adequately expressed. Your call upon me to respond for Virginia is so unexpected that I am unable to respond as I would like. But, gentlemen, in behalf of the Virginia dentists, allow me to say that, if you will give us the opportunity, we will welcome you to our State, to our "roof-trees, our hearth-stones, and our firesides," as well as to our hearts. My own desire is that, should you visit our old State, your every moment may be as pleasantly and profitably spent as my time in Boston has been. I thank you warmly for your kind expressions and for the cordial welcome you have extended me, in all of which I most heartily reciprocate.

*Dr. S. B. Palmer*, Syracuse, New York.—I consider it an honor to be appointed a delegate from the central part of New York, and I think there is something due this Society above the confession of that honor. I accepted the invitation to attend because I could not afford to stay away; and am thankful for being here, where we can obtain our supplies, as has already been referred to, and to accept your proffered hospitality and to know a fact which, as mentioned by the president, may be of some advantage to us,—that this Society is a committee of the whole to look after our interest and keep us from going astray. I thank you kindly for your courtesy.

*Dr. G. F. Cheney*, St. Johnsburg, Vermont.—I thank you very much for the invitation. You appear to be well represented, and everything points to an instructive and successful meeting. I had the pleasure of attending your meeting last summer, and the remembrance of it is a bright spot in the events of the year.

*Dr. Hayward*, New Hampshire.—A New Hampshire man would have known better than to call on me for any remarks. I had the pleasure of attending for the first time the Massachusetts Dental Society twenty-two years ago this month. At that time I was just entering your ranks as a student, and since then, although I have been plodding away among those New Hampshire hills, I have always been a student of the Massachusetts Dental Society. Your meetings have always been a source of profit to me, and I am heartily glad to be with you here to-day.

*Dr. J. Bond Littig*, New York City.—It is not necessary for me to say, Mr. President, that New York sends greetings. Our interests are closely allied, and you know, from visiting our societies in New York, that we are all friendly towards you. I know I get a good many good things here, and I expect to get a good many more things from Dr. Merriam.

*Dr. Geo. A. Maxfield*, Holyoke, Massachusetts.—I think I can say, at least for the members of our Society, that we are glad to attend this meeting, for only those who have labored in getting up these dental meetings know how much there is to do, and now this summer we have given up our semi-annual meetings, and are going to enjoy yours. I thank you heartily for the invitation.

*Dr. Frank Harrison*, Sheffield, England.—It may be interesting to you to know somewhat of the history of dentistry in England. I was present at many of the meetings of the Dental Reform Society when the great strife was being made to get dentistry recognized by our state. Up to within a few years ago any one could practise dentistry, without any degree whatever, but our Dental Diploma Committee managed to impress upon Parliament the necessity of some state recognition, and, accordingly, it was got with considerable modification. Of course, as you know, they had to recognize any one who had any claims. At the present time the Dentists' Register is giving us the greatest amount of trouble. At all our meetings we have placed before them the importance of clearing and keeping it correct.

Our Association meetings are divided into two sections,—a political and a scientific section. The political part of our meeting takes place first, and in that we discuss the general position of dentistry; those who should, and those who should not, be recognized; or any infringement of the "Dentist Act." Such, I presume, you have to a limited extent, although not quite so public; your meetings being of almost an entirely scientific character.

Another subject now coming to the front is the qualifications of young men,—whether they shall simply become dentists and then surgeons, or surgeons and then dentists. Some contend that the dental should be separate from the medical profession. Of course, our diploma is granted by the medical body—"Licentiate Dental Surgeon"—and not by dentists. Another topic is the formation of a higher degree in dentistry. In our colleges in England we have various degrees, and many consider that the degree of "L.D.S." is not sufficient; that there should be something higher for those who wish to distinguish themselves in dental studies; and that a distinction in dentistry should be made. That, I believe, will take much of the time of the annual meeting to be held at Brighton in August next. A man who is going to study surgery has to take up four years of his life in pursuing the curriculum and getting ready for his examination. In these four years he may also take up dental subjects, and obtain both the degree of M.R.C.S. and also the dental diploma; so we rather look forward to that as one thing that will place dentistry in England on a higher plane.

*Dr. Oswald Fergus*, Glasgow, Scotland.—I am very glad to be here and accept this invitation of your president to say a few words. You can scarcely imagine the pleasure I have in being back in America again.

At home, after the passage of the "Dentist Act," there was a great rush for registration, with an accompanying want of qualification. Before its passage a man might be working in a blacksmith-shop to-day and to-morrow be put on the register. Young men could go to certain licensing bodies and pass their examination and sign the curriculum without any college training or education whatsoever; yet dentistry was called at home a "learned profession." It seems to me that, if a diploma is worth anything, young men—no matter what their experience had been—should not be allowed to receive it without having gone through a prescribed curriculum of three or four years, as might be necessary.

With regard to this higher degree, we would very probably find the same thing occurring if we were able to get it,—that men who signed the curriculum would be put on the same footing as the higher degree men.

Another thing I am glad to see here is the practical part of the work. You gentlemen are very kind; you not only tell us how a thing is done, but you bring your patients here and show us how to do it; and if we cannot pick it up it is our fault and not yours. I had a good view of your clinics, and shall always be grateful to the members of the Society for the kindness shown me. I was an absolute stranger yesterday,—knew no one. Now I go back with many more friends than I thought possible to become acquainted with in so short a visit.

### DISCUSSION ON DR. PALMER'S PAPER, "ELEMENTS OF SUCCESS IN DENTAL PRACTICE."

*Dr. I. J. Weatherby*, Boston.—I was quite interested during the reading of the paper, and am in agreement with its general contents, but when he struck the vein of "copper amalgam" I was disturbed. It seems to me that we should have sufficient skill to save teeth without resorting to the blackness of darkness which is produced by copper amalgam. I am a positive man, and no one will take offence.

Some little time ago I was called upon to remove a copper amalgam filling from the mouth of a lady,—a superior right first bicuspid, mesial surface. I asked the patient why it was inserted. She told me that it was the dentist's choice. I said to her, "It is not always safe, perhaps, to leave the choice of matters to the dentist, in justice to yourself." The surface of the filling was black, and every time she opened her mouth there was a black sheep in the fold. It was removed, and all know how hard they are. It was drilled out, however; and when a gold filling was inserted she was delighted.

I have practised dentistry now for over forty years and have had no occasion to resort to the use of copper amalgam. You will remember, gentlemen, something like forty-five years ago the first society of American dentists organized put its foot down very severely on amalgam, inhibiting its use entirely; but they, of course, carried the matter too far. The amalgams of to-day are better than were the silver and quicksilver,—made by filing up a quarter,—which turned black, but preserved the teeth as well as copper amalgam.

Now, the introduction, in 1885, of copper amalgam is as much worse than Townsend's amalgam as this latter formula is worse than gold; and yet Townsend's was an improvement on the old amalgams. If skilfully used, the improved amalgams are a success, and are to a certain extent an improvement on Townsend's amalgam. In my opinion, the latter, even, is far preferable to copper amalgam. It is claimed that copper amalgam will preserve teeth that other amalgams will not. I assert that other amalgams will do the same thing. The main fault is in the manipulation. No patient with an amalgam filling leaves my office for an hour to an hour and a half after the filling has been inserted. The burnishing of the amalgam after it has crystallized is the great secret of success.

*Dr. S. B. Palmer*, Syracuse, New York.—I think I should know something about copper amalgam: I would not consider it right if I did not understand it, after the personal experiments I have made. The paper does not advocate it. I stated that eighty per cent. of carious teeth could be filled with gold. I think that is fair. The balance of twenty per cent. must be filled with something, and we do not know as yet whether copper amalgam is better than any other, or whether it is worse.

*Dr. G. L. Curtis*, Syracuse, New York.—Will Dr. Weatherby state why the filling was removed?

*Dr. I. J. Weatherby*, Boston.—Because it gave such a demoniacal character to the other teeth in the jaw that the lady was perfectly disgusted and horrified that she should receive such treatment from a dentist.

*Dr. G. L. Curtis*, Syracuse, New York.—I am glad, gentlemen, it was not because the tooth had decayed, and I therefore do not look upon the argument as a very strong one. Regarding the unsightliness of copper amalgam,—black ebony looks very pretty when polished. I think that we, as dentists, should practise with a view of preserving the teeth, and regard the appearance—the artistic effect—as a secondary consideration.

*Dr. L. D. Shepard*, Boston.—There is an element of success which the essayist has spoken of, but which he did not enlarge upon. I would like to present it in a little stronger light. I have noticed it in the experience of the last two years in examining men —mainly graduates of dental colleges—for license to practise dentistry in Massachusetts. The most noticeable feature has been an inability to reason correctly from conditions to results, and to adopt the proper treatment. I have been struck with the fact that young dentists—and, to a less extent, older dentists—do not receive what seems to me the fundamental principle of education,—a knowledge of how to reason, how to discuss the reason for a certain condition being as it is, and the best steps to take to secure success, and why. As an element to the highest success in practice, it seems to me that it deserves prominence, perhaps, above any other essential. Without enlarging upon it, I simply give it as my observation in the examination of between thirty and forty dentists in the past two years. Some of them received degrees from our best colleges, and some were in practice ten and twenty years. I shall enlarge upon this later on, and I consider it a very important point in dental education.

*Dr. J. H. Alexander*, Mystic River, Massachusetts.—It does seem that, with all the wise heads here, some one ought to give us some thought in reference to this amalgam question that would straighten out some of the knots that seem to be in our paths. This question has been discussed by many, and often in such a way as to be of little advantage. There is one thing that seems to operate upon our patients, and that is the manner in which we speak of other men's work. I think it is unjust to state to a patient that a thing is wrong unless we know it is absolutely wrong.

*Dr. F. A. Cooke*, Boston.—I am glad the question of copper amalgam has come up, and I am desirous of learning all that I can in reference to it. It should be argued for the benefit of the younger men, who are striving to do what is right, and they should have the benefit of the knowledge and experience of those who have been longest in the profession.

In a mild way I have taken to using copper amalgam,—using it according to directions, and according to the light I have so far received. I have made observations and tried to inform myself regarding it. I am glad to hear the idea of using a varnish over the dentine before inserting the filling, for all these things bring out points that I think should be known if there is value in them. There are one or two points I would like to be informed upon; and one is the electrical effect of copper amalgam coming in contact with a gold filling, and another is whether there is any constitutional effect coming from the chemical action in the mouth that will act deleteriously. I have had several patients lately—men learned in the sciences—that I have worked for, using copper amalgam; and I have felt called upon, before inserting the filling, to state that it was copper, and that it was being tried. They have closely questioned me sometimes, and to my disadvantage, having the advantage of me in their superior knowledge of chemistry; and I would be glad to receive any light on the subject you may have to offer.

*Dr. J. N. Crouse*, Chicago, Illinois.—The two great elements of success, in my opinion, are strict integrity and perseverance, coupled with the proper judgment of what seems wisest and best to do under the circumstances,—perseverance until the desired end is accomplished. The second point is the one brought out by Dr. Shepard,—a knowledge of the reason for a thing. A dentist ought to be logical. If I hear a man talking illogically on a subject, I know that if he is illogical in his reasoning he will be illogical in his practice. If you are going to have the brightest success, you must do the best for your patient first. If upon close observation you find your patients indifferent and careless in their habits, and you have performed a good operation that needs to be cared for, you must educate them to the necessity of cleanliness and try to elevate their standard in this respect, or your best work will prove a failure,—especially where there is a predisposition towards caries. I believe in contour-work where the part is decayed, or lost by caries, and to restore the shape of the tooth as originally formed by nature. If this is done, the patient can masticate food properly; whereas, if widely separated, the teeth, in many instances, are sources of discomfort. There is no comparison to be made between the two methods of operating for the comfort of the patient; but if they are not cleanly, and do not appreciate your efforts, your work will be in vain, and it scarcely matters what you fill the teeth with,—you will have constant failures.

The paper said that eighty per cent. could be filled with gold. I don't know that I would put it as high as that. Gold, however, stands pre-eminent as the superior of all other materials. It will save more teeth than anything else; but it requires the strictest integrity and determination to insert it properly. In preparing a cavity, if, after it is completed, you use a magnifying glass on it, and detect a little chalky line running across the cavity,—extending beyond where the cavity has formed, perhaps up the cervical margin,—you will have to remove this or you will have a failure.

Regarding the question of copper amalgam, it is too soon, I think, to give a positive opinion. In my opinion, it is the first amalgam brought to the attention of the profession that looks as though it would save teeth. I haven't used a great deal of it, but have watched it carefully. It is the first amalgam I have seen, that, after being inserted for a few years, has seemed to preserve the edges of the tooth perfectly. I had the pleasure of examining four or five patients of Dr. Dean's, and the fillings are standing beautifully. I think it is the coming amalgam. I have never seen any amalgam that looked very well for color, and do not recommend copper amalgam on that account. The time may come when we will consider ebony the prettiest color; but if I was a young man, and a young lady opened her mouth and showed her teeth filled with black amalgam,—well, I should have preferred that they were filled with gold.

Regarding gutta-percha, I have had the theory advanced to me that gutta-percha expands in teeth,—that, instead of shrinking, it enlarges. I have seen cases where it seemed to me that it did so. I throw this out as a query in order to get the experience of those who live where great knowledge comes from,—Boston,—get the experience of the men in this "land of knowledge" on this subject. I have seen pivot teeth set with gutta-percha, and it looked very much to me as if there was expansion of the gutta-percha. Some claim that it expands enough to split the tooth. I would like the experience of my friend Palmer and others on that subject.

*Dr. Charles S. Butler*, Buffalo.—I came to Boston to attend the meeting of the Massachusetts State Dental Society, expecting to find a great many things, but I did not expect to find the "color line" drawn so closely here as it has been. Perhaps, had our friend from South Carolina not left the room, he could have obtained some benefit on the color line in the subject of copper amalgam. I am very sorry that this copper amalgam, upon which the paper merely touched, should be singled out as the one question for discussion. There are many good points in the paper that are being lost sight of. One point was the earnestness of the essayist in treating the subject; and we can all emphasize the words of Dr. Crouse in regard to the necessity of application and earnestness in our profession, and the determination to find out what is the best thing. This is what the young men want, and it is a fundamental principle underlying all success. If I have anything to say in regard to copper amalgam, it is to query regarding its color,—is it much more unsightly than a highly-polished gold cap upon the first bicuspids? It does not seem to me to be so. Possibly our education will so advance that the time will come when we will think as well of the copper amalgam as we do now of the highly-polished gold caps.

*Dr. T. H. Parramore*, Hampton, Virginia.—I was much impressed with the point made regarding the examination of cavities after you have prepared them. Time and again I have found the imperfections, which cannot be discovered with the naked eye. Another point I think should be touched upon,—that of the thorough finishing of fillings after they are inserted, and here also we must have recourse to the magnifying glass. If we do not remove overlapping gold, we are bound to have failures, just as much as if we leave a jagged edge in the cavity. Run up closely under the margin, and see if any of the fine threads of floss silk will catch in the filling at the cervical wall. That is an important point in the filling of any approximal cavity.

I have experimented somewhat myself regarding this copper amalgam, but cannot say yet whether it is a proper thing or not. I am favorably impressed with it: it seems to adapt itself better to the walls of the cavity than any other amalgam I ever saw, whatever the results may be. I am waiting for results, and I think it will prove to be a great benefit to us as dentists, and also to our patients.

I take this occasion to say that I am very glad to be with the Massachusetts Dental Association.

*Dr. Oswald Fergus*, Scotland.—I have used copper amalgam for the past nine years, and found it to be a good filling material. In many of our hospitals this amalgam is much used. I do not know what superior it has. True, in cases where the filling is conspicuous its color is objectionable, but, if you will take cavities where the filling material is not seen,—in molars and wisdom-teeth,—I do not think much can be said against it. I would not, however, advise its use in every mouth. It should not be put in the hands of students and novices. The student should be taught to use gold, and if he can use this well he can work anything well.

*Dr. I. J. Weatherby*, Boston.—The color line here is very peculiarly drawn, as touching amalgam fillings. I do not care myself to confess to people that I cannot save teeth as well with gold as I can with amalgam. Any teeth you can save, that will bear filling with copper amalgam, I will fill with gold, and will continue the life of that tooth as long as with amalgam. I give you this challenge.

*Dr. Charles S. Butler*, Buffalo.—I had a patient that came to me ten or twelve years ago, with teeth that I could not fill with gold. I filled with amalgam, and stated that it might last three years, but how long was uncertain; these teeth are doing good service to-day, and I do not see why they should not last ten years more. I freely admit that I could not have done it with gold, but others might have done so.

*Dr. George A. Maxfield*, Holyoke, Massachusetts.—One thing regarding success, that has not been taken up, is the "money" part of the question. I say any man who will think of money first is not fit to belong to the dental profession, but it is a thing we all have to take into account. I do not think the essayist set the percentage at all high when he stated that eighty per cent. of the teeth could be filled and saved with gold. I think ninety per cent. would not have been at all too high. It takes skill, and skill can do it with gold, but with the majority of our profession their practice is not such that they can always use gold. While I was at college Dr. Webb was giving a clinic, and he made this remark to the students: "Do not, gentlemen, put in anything but gold, even if you have to pay for it yourselves." That, of course, was nonsense. A great many people in moderate circumstances come to us, and cannot afford high charges; yet it is our place to serve them, and what are we going to use? I answer, just that material that will make a good filling and save the teeth, and come in a line with their pocket-book.

I have used copper amalgam some, and experimented a great deal with it. I stated a year ago, when we discussed this subject, that Dr. Miller, one of our best workers, tells us copper amalgam is the only filling material around which germs will not live; and I think it is settled that caries of the teeth is caused by germs, or at least they are a factor. We want to do all we can to limit the development of organisms. We all find places in the mouth which the majority of persons do not keep clean,—approximal cavities in molars. In all such cases I place copper amalgam at the cervical border. It is said that copper amalgam does not unite with silver alloy. I am of that opinion myself. Last week I placed in a filling on the approximal surface of a second molar, and had to remove it yesterday because of inflammation of the pulp. I found my silver alloy came out with a clear ring from the copper. In using amalgam my patients cannot afford, and I cannot afford, to take the time that Dr. Weatherby does with an amalgam filling.

I am glad the discussion has taken the turn it has. It shows the deep interest in copper amalgam. It is coming into prominence, but a great many who are using it do not know how to manage it. Failures are the result, which should not be charged against the material itself.

*Dr. George F. Waters*, Boston, Massachusetts.—Of all men in the world, a company of dentists ought to understand the reason for a certain procedure. Why should copper amalgam be used, and what is the effect of it?

One evening, last winter, I was present at a meeting of the Boston Society of Natural History, and some facts that were presented seemed to have a bearing upon the question, and I reported the matter to the editor of the INTERNATIONAL JOURNAL; I think it was in the last number. A railroad had been laid from Boston down to one of our summer resorts, and workmen were at work cutting away the side of a hill. They came upon some Indian graves, and Prof. Putnam was sent for to see if there was anything worth saving. When he examined the graves he found Indian skeletons, interred there in such a way as to indicate that there had been some kind of an epidemic and that a large number had been destroyed.

In one of these graves was found a skeleton with a piece of copper bound over a portion of the head, and the integument, the hair, the skin, and the bone had been preserved,—no decomposition there, notwithstanding the rest of the skeleton was far gone. An important feature was that the brain was preserved and had shrunk up under the bone that was protected by the copper,—the copper salts having saturated it and passed through. The wrappings that were taken from that bone—the vegetable matter,—fibre, matwork material, etc.—were preserved, so that they have them now, showing the texture and method of making.

We all know that arable soil gets its fertile power from the ability of certain microbes to separate from the atmosphere nitrogen, and that when they come in contact with vegetable matter that matter is decomposed. It is arranged so as to serve the purpose of coming plants. What was the effect of this copper there in protecting these substances from the various low organisms about them? Here is a point for you to ponder on in regard to copper amalgam,—whether it has not the same power in the mouth. These skeletons had lain in that condition for more than two hundred years, and still, while substances that ordinarily decompose, they were preserved.

Subject passed.

*Thursday, June 6.—Afternoon Session.*

DISCUSSION ON PAPER, BY S. H. GUILFORD, D.D.S., PH.D.,—"PHYSIOLOGY OF TOOTH-MOVEMENT IN REGULATING."

*Dr. H. A. Baker*, Boston, Massachusetts.—To say I was interested in Dr. Guilford's paper does not express my feeling. There were two points touched upon about which too much cannot be said. One is the tendency of nature to do her own work. I believe if we give nature a chance she will do more towards regulating than we can do with all the appliances we in our ingenuity can make. If patients are taken when they are young enough, and the teeth trained as they are erupted, there is no need of regulating them. Nature will do the work.

Another subject of interest to me was the point of retaining teeth. This, I think, we should all consider. I have seen more trouble from retaining-plates than irregularities themselves; and if we can make fixed retaining appliances which will be cleanly, and, at the same time, fixed,—so the patient cannot remove them,—we will succeed much better. There is one little device which, so far as I know, is original with myself, and has proved of great value in retaining overlapping centrals after regulating: It consists of a little gold bar from one-eighth to three-sixteenths inch long. It is applied with oxyphosphate cement, and is a very simple and effective appliance. I have a case of four front teeth overlapping and out of line, and they are retained by this band alone.

*Dr. W. X. Sudduth*, Philadelphia.—The essayist stated that a cuspid forced itself into place. I do not think the tooth had the power in itself to accomplish the movement. The tooth was a passive object, and was forced into place by the action of the lip. The lip on the outside tends to constrict and force the teeth backward. The shape of the arch is largely due to the action of two forces. The tongue on the inside pressing out and the lips on the outside tending to constrict. The form of the tongue has much to do with shaping the arch. The restrictive action of the lips is also well known.

An illustration of the strength of the orbicularis oris muscle was shown in a case in my own practice some years ago, in a lady patient with overlapping central incisors. When she first came to me deep lines were noticed running down from the alæ of the nose, producing a pursed condition of the lips. Since then she died of a malignant disease,—carcinoma of the uterus. At that time she was evidently suffering more or less from the disease, and it gave her an expression of pain manifesting itself in the compression of the lips; and as a result of the constriction of the orbicularis muscle we had the teeth crowded. The arch was widened and a gold plate inserted as the only thing that would prevent a return of the original irregularity.

*Dr. S. C. G. Watkins*, Montclair, New Jersey.—I think the case which Dr. Sudduth speaks of is illustrative of other conditions than those of regulating teeth. I know the case of a child that has been under my care from birth. At birth and up to the third year the child had a perfectly-formed and normal arch; but it acquired the habit of breathing through its mouth. The result has been that the muscles, in the effort of holding the mouth open in that way, are drawn down over the teeth, and the arch is decreasing in width and becoming narrow, so much so that you cannot do more than place your finger in the centre of the arch: the child is now nine years of age. There has been a radical change in the shape of the mouth, and from no other reasons than mouth-breathing and the force of these muscles on the side of the mouth.

*Dr. Sudduth*, Philadelphia.—In this case, the mouth being held partially open, as is the case in all mouth-breathers, the expansive action of the tongue is lost, the tongue in such cases lying on the teeth, and nothing opposing the constrictive action of the lips, hence the narrow arch.

*Dr. H. A. Baker*, Boston.—Can Dr. Watkins explain any cause of the mouth-breathing?

*Dr. S. C. G. Watkins*, Montclair, New Jersey.—By an injury to the nose the cartilage was pressed in on one side, so that the nostril was almost closed, making breathing through the nose difficult. The child, not being old enough to know the necessity of persisting in the proper manner of breathing, adopted the easier mode, with the result as I have stated.

*Dr. T. H. Parramore*, Hampton, Virginia.—In my practice I have a patient, a boy thirteen years old, who has gotten into the habit of sucking his under lip. This pushes the lower teeth back and the upper teeth out, so that when he closes his mouth there is a distance of one-quarter to one-half inch between the upper and the lower teeth. I would be glad to know how to remedy it. He sleeps in that way, and, except when he is talking and eating, he is continually practising this habit. It has made the upper arch very sharp. I have tried to correct it, but have never been able to do any good.

*Dr. C. H. Hayward*, Peterborough, New Hampshire.—A short time ago I had a lady, one of whose canine teeth had erupted out of position, there not being sufficient space between the bicuspid and lateral incisor. She had never paid any attention to it, and was thirty years of age when she sought advice regarding it. Finally it got so bad that whenever she laughed her lip would catch up there and annoyed her exceedingly. I told her it was possible that it might come down if she would let me take out the bicuspid. I extracted it a year ago last March, and, with no force except that of the lips, this tooth has come down into position.

*Dr. I. J. Weatherby*, Boston.—Regarding the case mentioned by Dr. Parramore, I would take an heroic course. I would make a gold plate fitting upon the under teeth, pushing them out, and attach to the plate barbs, with which he might amuse his under lip at leisure. I think he would soon be cured. Any attempt at the old practice would be met by the stern sentence, "Thus far shalt thou go, and no farther."

Subject passed.

*Friday, June 7.—Morning Session.*

DISCUSSION ON PAPER BY E. A. BOGUE, M.D., NEW YORK CITY,—A "DESCRIPTION OF MAKING CASTS AND DIES, AND PROCESS OF SWEDGING."

*Dr. R. R. Andrews*, Cambridge, Massachusetts.—The screw-press for plates I have used for some time, and would not go back to the old way. I had mine made by a blacksmith, who charged me twelve dollars for it. Elsewhere, I suppose, it would have cost thirty dollars. If you experience the pleasure of getting a perfect fit, when the impression is all right, you will be well repaid for your trouble.

*Dr. E. G. Leech*, Boston.—I think for the past eighteen years I have never struck a blow on a gold plate to swedge it. The press I use is made of wrought iron, except the base. It is made with a screw, like that of Dr. Andrews,—of steel, engine-turned. In swedging a gold metal plate you cannot be as sure of results in any other way as with the press that carries it home without rebound, there being no resistance. On the last pressure place a thin piece of cotton cloth underneath your plate, as your first work will expand your counter-die, and the cloth makes up for expansion and makes a perfectly tight counter-die; your plate will be driven home, and a perfect adaptation the result.

Subject passed.

